# Supplementary material for: Chromatin accessibility during human first-trimester neurodevelopment
Source: Nature. 2024 May 1;647(8088):179–86. doi: 10.1038/s41586-024-07234-1 (PMC12589128; doi:10.1038/s41586-024-07234-1)
Supplement: Supplementary file 1 — Reporting Summary [file 41586_2024_7234_MOESM1_ESM.pdf]

nature portfolio

Corresponding author(s): Sten Linnarsson

Last updated by author(s): Jul 17, 2023

Reporting Summary

Nature Portfolio wishes to improve the reproducibility of the work that we publish. This form provides structure for consistency and transparency in reporting. For further information on Nature Portfolio policies, see our Editorial Policies and the Editorial Policy Checklist.

Statistics

For all statistical analyses, confirm that the following items are present in the figure legend, table legend, main text, or Methods section.

n/a	Confirmed	
☐	☒	The exact sample size (*n*) for each experimental group/condition, given as a discrete number and unit of measurement
☐	☒	A statement on whether measurements were taken from distinct samples or whether the same sample was measured repeatedly
☐	☒	The statistical test(s) used AND whether they are one- or two-sided<br>*Only common tests should be described solely by name; describe more complex techniques in the Methods section.*
☐	☒	A description of all covariates tested
☐	☒	A description of any assumptions or corrections, such as tests of normality and adjustment for multiple comparisons
☐	☒	A full description of the statistical parameters including central tendency (e.g. means) or other basic estimates (e.g. regression coefficient) AND variation (e.g. standard deviation) or associated estimates of uncertainty (e.g. confidence intervals)
☐	☒	For null hypothesis testing, the test statistic (e.g. *F*, *t*, *r*) with confidence intervals, effect sizes, degrees of freedom and *P* value noted<br>*Give P values as exact values whenever suitable.*
☒	☐	For Bayesian analysis, information on the choice of priors and Markov chain Monte Carlo settings
☐	☒	For hierarchical and complex designs, identification of the appropriate level for tests and full reporting of outcomes
☐	☒	Estimates of effect sizes (e.g. Cohen's *d*, Pearson's *r*), indicating how they were calculated

Our web collection on statistics for biologists contains articles on many of the points above.

Software and code

Policy information about availability of computer code

Data collection

10X Cellranger-ATAC 2.0.0
10X Cellranger-ARC 2.0.0

Data analysis

Programs:
Homer v4.11
bedtools v2.25.0

Python packages:

# Name	Version	Build	Channel
cytograph	2.0.1	dev_0	<develop>
deeplift	0.6.13.0	pypi_0	pypi
fisher	0.1.9	pypi_0	pypi
harmony-pytorch	0.1.4	pypi_0	pypi
loompy	3.0.6	dev_0	<develop>
macs2	2.2.7.1	pypi_0	pypi
milopy	0.1.1	pypi_0	pypi
modisco	0.5.16.2	dev_0	<develop>
numba	0.51.0	pypi_0	pypi
numpy	1.21.6	pypi_0	pypi
opentsne	0.4.4	pypi_0	pypi
pybedtools	0.8.1	pypi_0	pypi

```
pybigwig              0.3.17              pypi_0    pypi
pygam                 0.8.0               pypi_0    pypi
pynndescent           0.4.8               pypi_0    pypi
scikit-learn          0.23.2              pypi_0    pypi
statsmodels           0.12.1              pypi_0    pypi
ucsc-bedgraphtobigwig    377              h446ed27_1    bioconda
ucsc-bigwigaverageoverbed 377             h446ed27_1    bioconda
ucsc-liftover         447                 h954228d_0    bioconda
umap-learn            0.4.6               pypi_0    pypi
pytorch               1.12.1        py3.9_cuda11.6_cudnn8.3.2_0    pytorch
pytorch-lightning       1.7.7             pypi_0    pypi
pycistopic            1.0.2.dev9+gaf3977c        dev_0    <develop>
pyslingshot           0.0.2               pypi_0    pypi
skggm                 0.2.8               pypi_0    pypi
```

R:
ChromVAR 1.22.1

for GRN:
DELAY v0.1.0 https://github.com/calebclayreagor/DELAY
BoolODE v0.1 https://github.com/Murali-group/BoolODE

for GWAS enrichment:
LDSC v1.0.1 https://github.com/bulik/ldsc
MAGMA v1.0 https://ctg.cncr.nl/software/magma

All custom code for analysis is available through:
https://github.com/linnarsson-lab/fetal_brain_multiomics
https://github.com/linnarsson-lab/chromograph

For manuscripts utilizing custom algorithms or software that are central to the research but not yet described in published literature, software must be made available to editors and reviewers. We strongly encourage code deposition in a community repository (e.g. GitHub). See the Nature Portfolio guidelines for submitting code & software for further information.

Data

Policy information about availability of data

All manuscripts must include a data availability statement. This statement should provide the following information, where applicable:

- Accession codes, unique identifiers, or web links for publicly available datasets
- A description of any restrictions on data availability
- For clinical datasets or third party data, please ensure that the statement adheres to our policy

Data Availability. Raw sequencing data is available through from the European Genome Phenome Archive (EGAS00001007472). To facilitate ease of use of the resource the chromatin accessibility and gene expression data are browsable through the CATlas webbrowser (http://catlas.org/humanbraindev) and the convolutional neural network can be downloaded through github: https://github.com/linnarsson-lab/fetal_brain_multiomics.
Code Availability. All code used to reproduce the figures is available through github: https://github.com/linnarsson-lab/fetal_brain_multiomics. Code to reanalyze the data is available through: https://github.com/linnarsson-lab/chromograph. The DELAY models trained on scATAC-seq data are available through https://github.com/calebclayreagor/DELAY.

All our data is aligned to the GRCh38.p13 gencode V35 primary sequence assembly (https://www.gencodegenes.org/human/release_35.html)
We compared our data to the VISTA database (https://enhancer.lbl.gov) and the ENCODE cCRE datasets (https://www.encodeproject.org). We also used the HOCOMOCO transcription factor PWM database (https://hocomoco11.autosome.org). For the stratified LDSC analysis we made use of the UKBiobank (http://www.ukbiobank.ac.uk) and studies from the PGC (https://pgc.unc.edu).

Research involving human participants, their data, or biological material

Policy information about studies with human participants or human data. See also policy information about sex, gender (identity/presentation), and sexual orientation and race, ethnicity and racism.

Reporting on sex and gender

An equal number of male and female embryos were collected (13 male, 13 female) and used for all analysis in this manuscript. To correct for structural genomic differences between the sexes, X&Y chromosomal reads were disregarded during clustering. We found no clear sex differences in cell type abundances or sex-derived artifacts in the clustering. As such the findings in this paper refer to both sexes.

As our samples were collected and processed as soon as possible, the sex of the sample could not be determined ahead of time, as such we collected from both sexes and identified the sex based on the presence of Y-chromosomal reads in the sequence data. For each cell in the data matrices the assigned sex is available as a column attribute.

Gender was not considered in this manuscript as this is generally considered a post-natal phenomenon.

Reporting on race, ethnicity, or

Samples were collected anonymously from donation in Sweden and the United Kingdom. The donation procedures did not allow us to collect race or ethnicity or similar information about the donors. As such we do not know if the ethnic

other socially relevant groupings	backgrounds of the samples are broadly representative of the general population, but they are likely to skew towards a western/northern European Caucasian background.
Population characteristics	Samples were collected only from healthy abortions, meaning there was no medical reason to the end the pregnancy. No chromosomal aberrations were detected in the clinic for these samples nor were any other diagnoses made.
Recruitment	Donors were recruited after electing to proceed with voluntary termination of pregnancy. As we do not have access to personal information of the parents, we do not know if there a self-selection bias in the data. In short, there might be differences between different parts of the public in their propensity to donate to science. For instance, there might be a relative over-representation of education level of the mother among our samples compared to the total of performed abortions for that reason. However, we do not expect this to impact the data in a strong way.
Ethics oversight	For UK, by the National Research Ethics Committee East of England, Cambridge Central and the North East – Newcastle & North Tyneside 1 Research Ethics Committee (DNR2019-04595); for Sweden by Etikprövningsmyndigheten (DNR2020-02074).

Note that full information on the approval of the study protocol must also be provided in the manuscript.

Field-specific reporting

Please select the one below that is the best fit for your research. If you are not sure, read the appropriate sections before making your selection.

☒ Life sciences ☐ Behavioural & social sciences ☐ Ecological, evolutionary & environmental sciences

For a reference copy of the document with all sections, see nature.com/documents/nr-reporting-summary-flat.pdf

Life sciences study design

All studies must disclose on these points even when the disclosure is negative.

Sample size	Sample size was dictated by the availability of scarce early developmental human samples, and based on prior experience with similar studies in mice. No power calculations were performed to determine sample size.
Data exclusions	Data for individual cells was excluded based on quality control metrics detailed in the manuscript (Methods)
Replication	The reproducibility of the dataset across specimens was assessed by assessing the contribution of donors to each cluster (Supplemental Fig. 1d). The LDSC analysis linking Major Depressive Disorder to Midbrain GABAergic neurons was validated in a separate GWAS cohort.
Randomization	Not applicable, as we did not perform any treatment vs control experiments.
Blinding	The investigators were not blinded, as this was an exploratory study with anonymous untreated samples.

Reporting for specific materials, systems and methods

We require information from authors about some types of materials, experimental systems and methods used in many studies. Here, indicate whether each material, system or method listed is relevant to your study. If you are not sure if a list item applies to your research, read the appropriate section before selecting a response.

Materials & experimental systems

n/a	Involved in the study
☒	☐ Antibodies
☒	☐ Eukaryotic cell lines
☒	☐ Palaeontology and archaeology
☒	☐ Animals and other organisms
☒	☐ Clinical data
☒	☐ Dual use research of concern
☒	☐ Plants

Methods

n/a	Involved in the study
☒	☐ ChIP-seq
☒	☐ Flow cytometry
☒	☐ MRI-based neuroimaging
